# Dentition

**Published:** 1890-10

**Authors:** 


### Dentition.

The cutting of teeth give rise to local and general symptoms and which demand both local and general treatment.

The druling of babies, when copious, may be controlled by small doses of belladonna. A teething child likes to press its gums again hard substances. For this purpose a rubber ring or a German pretzel will answer well. If a tooth is nearly through and the gum is tense over it a free crucial incision will frequently alarming reflex symptoms.

If there is gingivitis scarifying the gum by light touches of the lancet will lessen the hyperæmia and afford some relief.

For the moist eruptions about the head and face apply oxide of zinc ointment, black-wash, etc., and if you can always effect a cure before the teeth that caused it are through, your success will be greater than mine has been.

In the general treatment you will frequently be importuned to prescribe for a condition of feverishness, nervous erethism, and fretfulness that makes many a teething child a terror to the household. Here the bromides will render good service. I commonly give from two to five grains in solution with syrup flavored with peppermint or wintergreen, repeating as may seem necessary. If the infant is over-wakeful an equal quantity of chloral may be given in similar solution. At bedtime it is my habit to order a hot foot-bath, and a grain or two of Dover's powder. I may here say that the Dover's that I use with children is made with potassium bromide in place of the inert potassium sulphate. Do not neglect to avail yourselves of the power that aconite has to reduce fever. Give it in small doses, but repeat often. Add from five to twelve drops of the tincture to a full goblet of water. Give a teaspoonful every fifteen minutes for two hours; then every hour.

As to the diarrhœa that so often attends dentition in the hot months, there is a wide-spread belief among the laity that it is salutary and need not be interfered with. We may, perhaps, allow that a moderate looseness tends to prevent cerebral hyperæmia and some of the unpleasant reflex symptoms, yet it must not be forgotten that a profuse diarrhœa with dentition is as exhausting and as certainly fatal, if not checked, as though due to any other cause. So, if the movements should exceed three or four in the day it will be well to try to limit their over-frequency. The particular means will be mentioned when we come to speak of summer diarrhœa. You will have occasion to notice that a flux from teething is less amenable to treatment than that from some other causes, as over-eating. The "tooth cough" also is apt to be rebellious under treatment. Often you will succeed better with remedies that are sedative to the nervous system, than with the ordinary expectorants.

When an infant of teething age has convulsions and the cause is not evident, always examine the mouth. If you find the gum tense over a crown that can be plainly felt or seen, there can be no harm in making a crossed incision through it. Possibly you may thereby lessen the tendency to fits. Very generally, however, you will need to resort to other treatment, as the hot bath and the bromides, with or without chloral. When there are threatenings of convulsions it is my habit to treat them with a light dose—one to three grains—of calomel, or hydrargyrum cum creta—two to five grains—with about the same quantity of powdered rhubarb, or followed after some hours by a dose of castor oil or castoria. Besides that I give one of the bromides in such doses, and at such intervals, as may be necessary to control the convulsive tendencies.

If you meet with annoying urinary symptoms as a result of dentition, you will, I think, get more assistants from the bromides, either alone or with chloral, than from diuretics.

During the whole period of the first dentition particular care should be given to the diet, because the child is then, more than at other times, prone to digestive disorders.

The second dentition is generally accomplished with so little local and systemic perturbation as to need no treatment, though the wisdom-teeth, as some of us will remember, are capable of causing "trouble and sorrow."—Dr. Wm. T. Plaut, *Archives of Pediatrics.*

